# *Lactobacillus gasseri* NK109 and Its Supplement Alleviate Cognitive Impairment in Mice by Modulating NF-κB Activation, BDNF Expression, and Gut Microbiota Composition

**DOI:** 10.3390/nu15030790

**Published:** 2023-02-03

**Authors:** Soo-Won Yun, Hee-Seo Park, Yoon-Jung Shin, Xiaoyang Ma, Myung Joo Han, Dong-Hyun Kim

**Affiliations:** 1 Department of Food and Nutrition, Kyung Hee University, Seoul 02447, Republic of Korea; 2 Neurobiota Research Center, College of Pharmacy, Kyung Hee University, Seoul 02447, Republic of Korea

**Keywords:** *Lactobacillus gasseri*, aging, dementia, fecal microbiota transplantation, gut microbiota dysbiosis, gut inflammation

## Abstract

Aging-related gut microbiota dysbiosis initiates gut inflammation and microbiota dysbiosis, which induce the occurrence of psychiatric disorders including dementia. The alleviation of gut microbiota dysbiosis by probiotics is suggested to be able to alleviate psychiatric disorders including cognitive impairment (CI). Therefore, to understand how probiotics could alleviate CI, we examined the effects of anti-inflammatory *Lactobacillus gasseri* NK109 and its supplement (NS, mixture of NK109 and soybean embryo ethanol extract) on cognitive function in aged (Ag), 5XFAD transgenic (Tg), or mildly cognition-impaired adult fecal microbiota (MCF)-transplanted mice. Oral administration of NK109 or NS decreased CI-like behaviors in Ag mice. Their treatments suppressed TNF-α and p16 expression and NF-κB-activated cell populations in the hippocampus and colon, while BDNF expression was induced. Moreover, they partially shifted the β-diversity of gut microbiota in Ag mice to those of young mice: they decreased Bifidobacteriaceae, Lactobacillaceae, and Helicobacteriaceae populations and increased Rikenellaceae and Prevotellaceae populations. Oral administration of NK109 or NS also reduced CI-like behaviors in Tg mice. Their treatments induced BDNF expression in the hippocampus, decreased hippocampal TNF-α and Aβ expression and hippocampal and colonic NF-κB-activated cell populations. NK109 and NS partially shifted the β-diversity of gut microbiota in Tg mice: they decreased Muribaculaceae and Rhodospiraceae populations and increased Helicobacteriaceae population. Oral administration of NK109 or NS decreased MCF transplantation-induced CI-like behaviors in mice. NK109 and NS increased hippocampal BDNF expression, while hippocampal and colonic TNF-α expression and NF-κB-activated cell populations decreased. These findings suggest that dementia can fluctuate the gut microbiota composition and NK109 and its supplement NS can alleviate CI with systemic inflammation by inducing BDNF expression and suppressing NF-κB activation and gut microbiota dysbiosis.

## 1. Introduction

Aging is a progressive, degenerative process tightly associated with chronic inflammation, which can develop complex diseases such as dementia, sarcopenia, gut inflammation, and gut microbiota dysbiosis [1,2,3]. Moreover, patients with a psychiatric disorder have co-morbid inflammatory bowel disease and gut microbiota dysbiosis [4,5].

A variety of stressors including pathogen infections, antibiotics, and ageing cause the loss of beneficial gut microbiota and overexpression of gut bacterial endotoxins, resulting in gut microbiota dysbiosis, which can cause gut inflammation [6,7,8,9]. Gut microbiota-mediated gut inflammation is closely connected with the occurrence of dementia through the down-regulation of NF-κB activation-involved brain-derived neurotropic factor (BDNF) expression [8,10,11]. Thus, psychiatric disorders can cause gut inflammation and gut microbiota dysbiosis, which cause deterioration in psychiatric disorders AGAIN. Gut microbiota are bidirectionally connected with the brain through microbiota–gut–brain (MGB) hypothalamus–pituitary–adrenal (HPA) axes [12,13,14]. Moreover, anti-inflammatory *Bifidobacterium longum* NK46 alleviates cognitive decline in aged (Ag) or 5xFAD-transgenic mice [15]. *Lactobacillus plantarum* C29 mitigates cognitive decline in 5xFAD or Ag rodents by down-regulating NF-κB activation [16,17,18]. These findings suggest that anti-inflammatory probiotics may improve cognitive impairment (CI). *Lactobacillus gasseri* NK109 alleviates *Escherichia coli*-induced CI with gut microbiota dysbiosis [19]. *Lactobacillus paracasei* PS23 improves cognitive decline in D-galactose-induced aging mice by the regulation of the gut microbiome [20]. *L. plantarum* DP189 alleviates D-galactose/AlCl_3_-induced CI in mice by modulating gut microbiota [21]. Anti-inflammatory *Lactobacillus plantarum* OLL2712 improves cognitive decline in older adults by the attenuation of gut microbiota dysbiosis [22]. These findings suggest that gut microbiota dysbiosis-ameliorating probiotics may alleviate CI. The combination of *Lactobacillus casei* and dietary fiber alleviates CI brain function in Ag mice [23]. *L. plantarum* C29-supplemented DW2009 also increases cognitive function in volunteers with mild cognitive impairment (MCI) [24]. These results suggest that gut microbiota dysbiosis-attenuating probiotics and their supplements may be beneficial for the treatment of dementia.

Therefore, to understand how probiotics could alleviate dementia, we investigated the effect of *L. gasseri* NK109 and its supplement (NS) on cognitive function in Ag, 5xFAD transgenic (Tg), or mild cognition-impaired aged adult fecal microbiota (MCF)-transplanted mice.

## 2. Materials and Methods

### 2.1. Materials

De Man, Rogosa, and Sharpe (MRS) medium was purchased from BD (Franklin Lakes, NJ, USA). A soybean embryo ethanol extract powder (SE) was purchased from Mirae Biotech (Pocheonshi, Gyunggi-do, Republic of Korea).

### 2.2. Culture of L. gasseri NK109

NK109 was cultured in commercial media for probiotics, including MRS broth at 37 °C for 10 h, centrifuged, and freeze-dried [19]. NK109-contained supplement NS was prepared by mixing freeze-dried NK109 (0.5 × 10^10^ colony-forming unit [CFU]) with SE (10 mg), which was dry-heated at 60 °C for 30 min with shaking. NK109 and NS were suspended in saline for in vivo experiment.

### 2.3. Volunteers

Healthy volunteers (62–70 yrs old, 74–77 in CERAD-K, *n* = 3 [male, 1; female, 2]) and volunteers with MCI (62–70 yrs old, 52–63 in CERAD-K, *n* = 3 [male, 1; female, 2]) were recruited from Kyung Hee University Hospital (Seoul, Republic of Korea) (Supplementary Table S1). All volunteers enrolled for the stool collection are described in detail in Supplement File S1.

### 2.4. Animals

Ag C57BL/6 mice (male, 18-month-old), Tg C57BL/6 mice ((male, 4-month-old), adult C57BL/6 mice (male, 4-month-old), and young (Yg) C57BL/6 mice (male, 6-week-old) were purchased from RaonBio Co., Ltd. (Yongin-Shi, Republic of Korea). Mice were maintained in a controlled room and fed with water and food ad libitum. Animals were acclimatized for 7 days and used in the experiments. All animal experiments were ethically approved by the Committee for the Care and Use of Laboratory Animals in the University (IACUC, KHUASP(SE)-22129, 22236, and 22359) and were performed according to the Ethical Policies and Guidelines of Kyung Hee University for Laboratory Animals Care and Use.

First, mice were randomly divided in three groups of Ag mice (Ag, AgN, and AgS) and one group of Yg mice (Yg), consisting of six mice per group. Test agents (Yg, saline; Ag, saline; AgN, 1 × 10^9^ CFU NK109 per mouse; and AgS, NS [0.5 × 10^9^ CFU NK plus 1 mg SE] per mouse) were orally gavaged once a day (6 times per week) for 8 weeks.

Second, mice were randomly divided in three groups of Tg mice (Ag, AgN, and AgS) and one group of adult mice (Ad), consisting of seven mice per group. Test agents (Ad, saline; Tg, saline; TgN, 1 × 10^9^ CFU NK109 per mouse; and TgS, NS [0.5 × 10^9^ CFU NK plus 1 mg SE] per mouse) were orally gavaged once a day (6 times per week) for 8 weeks.

Third, mice were randomly divided in three groups of MCF-transplanted mice (Fm, FmN, and FmS), one group of healthy volunteer fecal microbiota (HF)-transplanted one group (Fh), and one group of Yg mice (Yg), consisting of seven mice per group. Cognition-impaired mice were prepared by oral gavage of MCF once a day for 5 days. Test agents (Yg, saline; Fh, saline; FmN, 1 × 10^9^ CFU NK109 per mouse; and FmS, NS [0.5 × 10^9^ CFU NK plus 1 mg SE] per mouse) were orally gavaged once a day for 5 days after the final transplantation of MCF or HF.

Cognitive function was measured in the Y-maze task (YMT), novel object recognition task (NORT), or Barnes maze task (BMT) the next day after the final gavage of test agents. Mice were euthanized in a CO_2_ chamber, followed by cervical dislocation.

### 2.5. Behavioral Tasks

YMT, NORT, and BMT were performed in a three-arm horizontal maze (40 cm long and 3 cm wide with 12-cm-high walls), open field box (45 × 45 × 45 cm), and circular platform (89 cm, diameter) with 20 holes (5 cm, diameter)/one escape hole box, respectively, as previously reported [19,25]. The detailed protocols are described in Supplement.

### 2.6. Immunblotting and Enzyme-Linked Immunosorbent Assay (ELISA)

The brain and colon tissues were lysed in radio immunoprecipitation assay lysis buffer (Pierce, Rockford, IL, USA) and centrifuged (10,000× *g*, 4 °C, 10 min). In the supernatant, BDNF, p16, p65, p-p65, CREB, p-CREB, Presenilin (Psen)-1, amyloid-β (Aβ), claudin-5, claudin-1, and β-action expression levels were assayed by immunoblotting [15]. IL-1β, IL-10, and TNF-α levels were determined using commercial ELISA kits (Ebioscience, Waltham, MA, USA) [15]. The detailed protocols are described in Supplement File S1.

### 2.7. Immunofluorescence Assay

Transcardially perfused brain and colon tissues were sectioned, as previously reported [23]. The sections were immunostained with primary antibodies against NeuN, BDNF, NF-κB, LPS, Iba1, and/or CD11c overnight and secondary antibodies conjugated with Alexa Fluor 594 (1:200, Invitrogen, Waltham, MA, USA) or Alexa Fluor 488 for 2 h. Immunostained sections were observed using a confocal microscope.

### 2.8. Microbiota Sequencing

Bacterial genomic DNAs were extracted from the fresh stools of volunteers using a QIAamp DNA stool mini kit (Qiagen, Hielden, Germany) and amplified using barcoded primers (bacterial 16S rRNA V4 gene region 16S rRNA genes) [26]. The amplicon sequencing was performed using Illumina iSeq 100. Sequenced reads were deposited in the short read archive of NCBI (accession number PRJNA 915132).

### 2.9. Quantitative Real-Time Polymerase Chain Reaction (qPCR)

qPCR for NK109 was performed on the Rotor-Gene Q^®^ thermocycler using DNA polymerase and SYBR Green I (Takara Bio Inc., Kusatsu, Japan), as previously reported [27].

### 2.10. Statistical Analysis

Experimental data are indicated as mean ± SD using GraphPad Prism 9 (GraphPad Software, Inc.). The significant difference was analyzed using one-way ANOVA followed by Duncan’s multiple range test (*p* < 0.05). The β-diversity (principal coordinate analysis [PCoA] plot) was indicated on the basis of generalized-unifrac as distance metric. The network of differentially enriched gut microbiota in spontaneous alteration in YMT or exploration in NORT scores was indicated on the basis of Pearson correlation coefficient (−1 ~ −0.22/0.22 ~ 1).

## 3. Results

### 3.1. NK109 Alleviated MCF-Induced Cognitive Impairment (CI) in Mice

**Figure 1:**
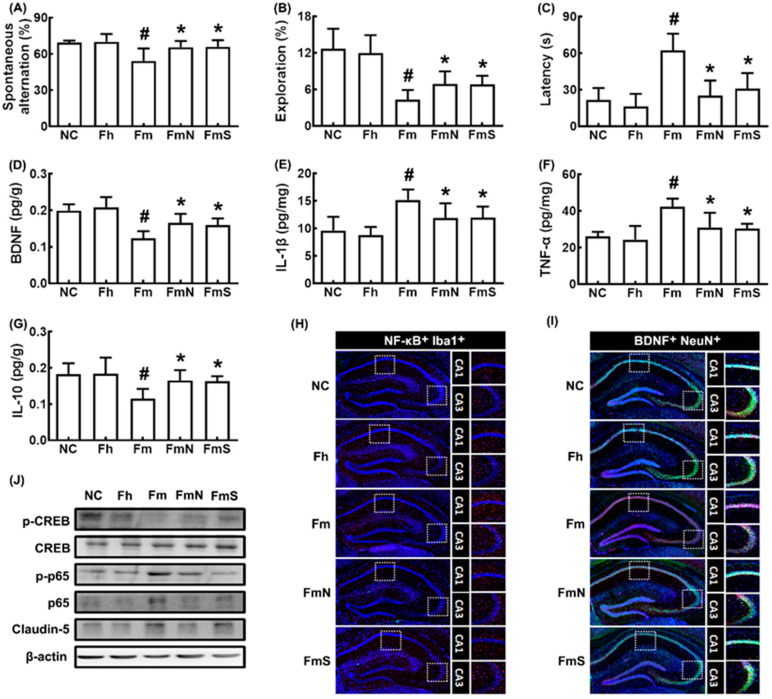
Effects of NK109 and its supplement NS on CI in MCF-transplanted mice. Effects on CI-like behaviors in the YMT (**A**) and NORT (**B**), and BMT (**C**). Effects on BDNF (**D**), IL-1β (**E**), TNF-α (**F**), and IL-10 (**G**) expression in the hippocampus. Effects on NF-κB^+^Iba1^+^ (**H**) and BDNF^+^NeuN^+^ cell populations (**I**) in the hippocampus. (**J**) Effects on the expression of p-CREB, CREB, p-p65, p65, claudin-5, and β-action. NC, normal control mice; Fh, HF-transplanted mice; Fm, MCF-transplanted mice; FmN, treated with NK109 in MCF-transplanted mice; FmS, treated with NS in MCF-transplanted mice. Data values are indicated as mean  ±  SD (*n* = 6). ^#^ *p*  <  0.05 vs. Fh. * *p*  <  0.05 vs. Fm.

First, we examined whether the MCF microbiota transplantation could cause cognitive impairment in mice (Figure 1). Orally gavaged MCF microbiota impaired cognitive behaviors in the YMT, NORT, and BMT to 76.9% (F_4,30_ = 8.21, *p* < 0.001), 29.8% (F_4,30_ = 2.99, *p* < 0.001) and 374.0% (F_4,30_ = 18.27, *p* < 0.001) of control mice, respectively, while orally gavaged HF microbiota were not affected (Figure 1A–C). However, the average of the arm entry numbers in the Y-maze task were not significantly different between all tested groups, suggesting that MCF treatment did not affect general locomotor activity, as described in the experiment of Sarter et al. [28]. To understand whether MCF could affect the expression of cognition-associated neuroinflammation makers TNF-α and IL-1β and cognitive function-inducing biomarkers BDNF [8,11], these biomarkers were assayed in the hippocampus using ELISA kits. MCF transplantation increased TNF-α and IL-1β expression in the hippocampus, while BDNF expression was decreased (Figure 1D–G). Next, we investigated the effects of NK109 and NS on MCF-impaired CI in mice (Figure 1A–C). Oral administration of NK109 and its supplement NS increased spontaneous alteration in the YMT to 94.3% and 94.9% of control mice, respectively, and exploration in the NORT to 47.7% and 50.0% of control mice, respectively, and decreased latency time in the BMT to 132.3% and 161.7% of control mice, respectively. Furthermore, their treatments decreased IL-1β and TNF-α expression, NF-κB^+^Iba1^+^ (activated microglial) cell numbers in the hippocampus, while BDNF, claudin-5, and IL-10 expression and BDNF^+^NeuN^+^ cell numbers were increased (Figure 1D–I). In the immunoblotting analysis, they also decreased p-p65/p65 expression in the hippocampus, while p-CREB/CREB and claudin-5 expression increased (Figure 1J).

**Figure 2:**
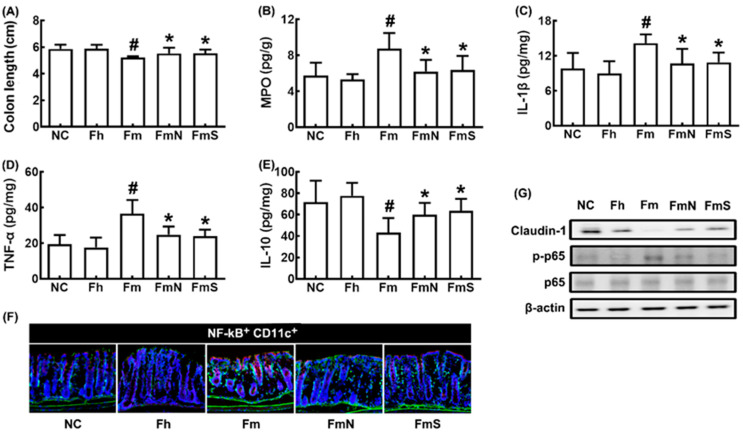
Effects of NK109 and its supplement NS on colitis in MCF-transplanted mice. Effects on colon length (**A**) and myeloperoxidase (MPO, **B**), IL-1β (**C**), TNF-α (**D**), and IL-10 (**E**) expression in the colon, assessed by ELISA. (**F**) Effects on NF-κB^+^CD11c^+^ cell population in the colon, assessed by a confocal microscope. (**G**) Effects on the expression of claudin-1, p-p65, p65, and β-action, assessed by immunoblotting. NC, treated with saline in normal control mice; Fh, treated with saline in HF-transplanted mice; Fm, treated with saline in MCF-transplanted mice; FmN, treated with NK109 in MCF-transplanted mice; FmS, treated with NS in MCF-transplanted mice. Data values are indicated as mean  ±  SD (*n* = 6). ^#^ *p*  <  0.05 vs. Fh. * *p*  <  0.05 vs. Fm.

MCF transplantation shortened colon length and increased IL-1β, TNF-α, and myeloperoxidase expression and NF-κB^+^CD11c^+^ cell numbers in the colon, while IL-10 and claudin-1 expression decreased (Figure 2A–F)). However, treatment with NK109 or its supplement NS down-regulated MCF-induced myeloperoxidase, TNF-α, and IL-1β expression and up-regulated claudin-1 and IL-10 expression.

### 3.2. NK109 and Its Supplement NS Improved Cognitive Function and Neuroinflammation in Ag Mice

**Figure 3:**
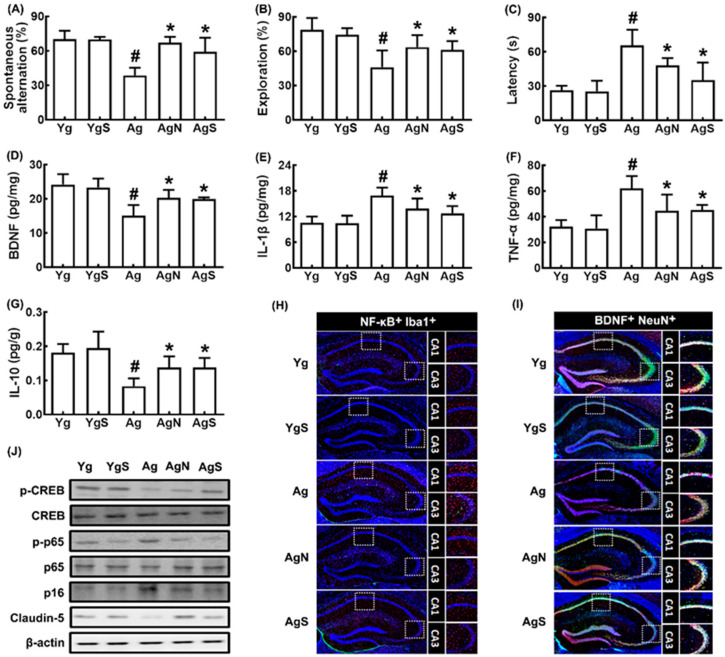
Effects of NK109 and its supplement NS on CI in Ag mice. Effects on CI-like behaviors in the YMT (**A**) and NORT (**B**), and BMT (**C**). Effects on BDNF (**D**), IL-1β (**E**), TNF-α (**F**), and IL-10 (**G**) expression in the hippocampus. Effects on NF-κB^+^Iba1^+^ (**H**) and BDNF^+^NeuN^+^ cell populations (**I**) in the hippocampus. (**J**) Effects on the expression of p-CREB, CREB, p-p65, p65, p-16, claudin-5, and β-action. Yg, treated with saline in young mice; YgS, treated with NS in Yg mice; Ag, treated with saline in Ag mice; AgN, treated with NK109 in Ag mice; AgS, treated with NS in Ag mice. Data values are indicated as mean  ±  SD (*n* = 7). ^#^ *p*  <  0.05 vs. Yg. * *p*  <  0.05 vs. Ag.

We also investigated the effect of NK109 and its supplement NS on cognitive function in aged mice (Figure 3). Ag mice showed significant deficits in learning and memory function compared to Yg mice: they decreased spontaneous alteration in the YMT and exploration in the NORT to 54.5% (F_4,30_ = 26.97, *p* < 0.001) and 58.0% (F_4,30_ = 13.02, *p* < 0.001) of Yg mice, respectively, and increased latency time to 247.5% (F_4,30_ = 25.32, *p* < 0.001) of Yg mice (Figure 3A–C). However, oral administration of NK109 and NS improved spontaneous alteration in Ag mice to 95.7% and 84.3% of Yg mice, respectively, exploration in Ag mice to 80.7% and 77.5% of Yg mice, respectively, and latency time in Ag mice to 180.1% and 127.7% of Yg mice, respectively. The cognition-improving potency between NK109 and its supplement NS was not significantly different. The expression of p16, TNF-α, and IL-1β expression and population of NF-κB^+^Iba1^+^ cell (activated microglia) population were higher in Ag mice than in Yg mice, while claudin-5 expression and BDNF^+^NeuN^+^ cell population increased (Figure 3D–I). In the immunoblotting analysis, they increased p-CREB/CREB and claudin-5 expression, while p-p65/p65 and p16 expression were decreased (Figure 3J).

**Figure 4:**
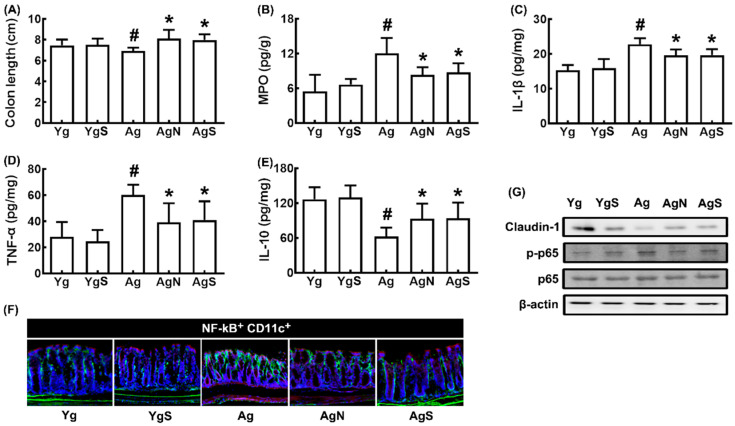
Effects of NK109 and its supplement NS on colitis in Ag mice. Effects on colon length (**A**) and myeloperoxidase (MPO, **B**), IL-1β (**C**), TNF-α (**D**), and IL-10 (**E**) expression in the colon. (**F**) Effects on NF-κB^+^CD11c^+^ cell population in the colon. (**G**) Effects on the expression of claudin-1, p-p65, p65, and β-action. Yg, treated with saline in young mice; YgS, treated with NS in Yg mice; Ag, treated with saline in Ag mice; AgN, treated with NK109 in Ag mice; AgS, treated with NS in Ag mice. Data values are indicated as mean  ±  SD (*n* = 7). ^#^ *p*  <  0.05 vs. Yg. * *p*  <  0.05 vs. Ag.

In Ag mice, colon shortening and colonic myeloperoxidase, TNF-α, and IL-1β expression and NF-κB^+^CD11c^+^ cell numbers were increased more strongly than in young control mice, while IL-10 and claudin-1 expression decreased (Figure 4A–G). However, treatment with NK109 or NS down-regulated myeloperoxidase, TNF-α, and IL-1β expression and up-regulated IL-10 and claudin-1 expression in the colon of Ag mice.

**Figure 5:**
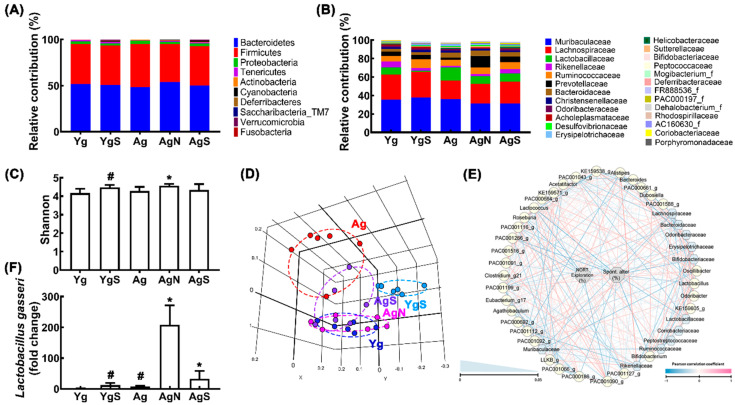
Effects of NK109 and NS in the gut microbiota composition in Ag mice. Effects on the composition at the phylum level (**A**) and family level (**B**). Effects on the α-diversity (OTU richness, c) (**C**), β-diversity (principal coordinate analysis plot based on Jensen–Shannon analysis) (**D**), and network of differentially enriched gut microbiota in spontaneous alteration in YMT or exploration in NORT scores (**E**). (**F**) Effects on the NK109 population. ^#^ *p*  <  0.05 vs. Nc. * *p*  <  0.05 vs. Ag.

We compared the fecal microbiota composition of aged mice with that of Yg mice (Figure 5, Supplementary Tables S2–S4). Ag mice exhibited a higher abundance of Proteobacteria and Actinobacteria populations compared to those in Yg mice (Figure 5A,B). However, the β-diversity between them was significantly different, while the α-diversity was not (Figure 5C,D). However, oral administration of NK109, but not NS, increased α-diversity in Ag mice (Figure 5C). Treatment with NS increased α-diversity in Yg mice. Nevertheless, treatment with NK109 or NS partially shifted β-diversity in Ag mice to that in Yg mice (Figure 5D). At the phylum level, treatment with NK109 or NS increased Tenericutes, Cyanobacteria, and Deferribacteres populations in Ag mice, while Actinobacteria population decreased in NK109-treated mice alone (Figure 5A, Supplementary Tables S3–S5). At the family level, treatment with NK109 or NS decreased Lactobacillaceae and Bifidobacteriaceae populations, while Rikenellaceae, Prevotellaceae, and Deferribacteraceae populations increased. In particular, treatment with NK109 alone decreased Helibacteriaceae and Odorobacteriaceae populations, while Porphyromonacaceae, FR888536_f populations increased (Figure 5B). In the network analysis, Bifidobacteriaceae, Lactbacillaceae, Bacteroidaceae, and Erysipelotrichaceae populations were negatively associated with spontaneous alteration in the YMT or exploration in the NORT, while Lachnospiraceae and Rikenellaceae populations were positively associated (Figure 5E, Supplementary Tables S6 and S7). Moreover, NK109 or NK treatment increased NK109 population in the feces of Ag mice (Figure 5E).

### 3.3. NK109 and Its Supplement NS Improved Cognitive Function and Neuroinflammation in Tg Mice

**Figure 6:**
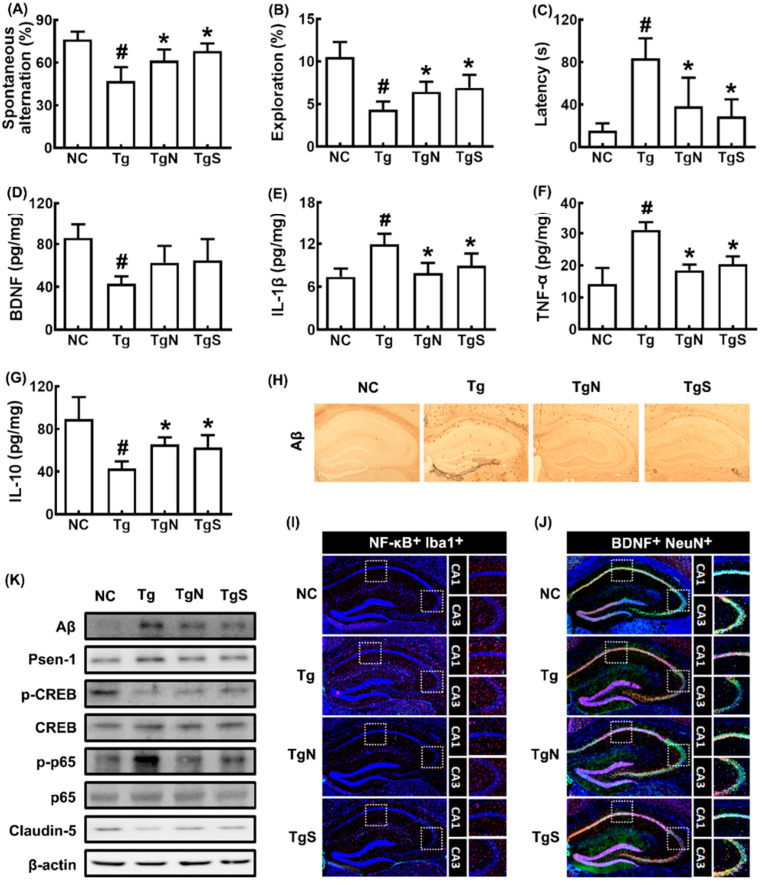
Effects of NK109 and its supplement NS on CI in 5xFAD transgenic (Tg) mice. Effects on CI-like behaviors in the YMT (**A**), NORT (**B**), and BMT (**C**). Effects on BDNF (**D**), IL-1β (**E**), TNF-α (**F**), and IL-10 (**G**) expression in the hippocampus. Effects on Aβ expression (**H**), NF-κB^+^Iba1^+^ (**I**) and BDNF^+^NeuN^+^ cell populations (**J**) in the hippocampus. (**K**) Effects on the expression of Aβ, Psen-1, p-CREB, CREB, p-p65, p65, claudin-5, and β-action. NC, normal control mice; Tg, treated with saline in Tg mice; TgN, treated with NK109 in Tg mice; TgS, treated with NS in Tg mice. Data values are indicated as mean  ±  SD (*n* = 7). ^#^ *p*  <  0.05 vs. Nc. * *p*  <  0.05 vs. Tg.

We also investigated the effects of NK109 and NS on CI in Tg mice (Figure 6). Tg mice exhibited significant deficits in learning and memory function compared with control mice: they decreased spontaneous alteration and exploration to 61.5% (F_3,20_ = 18.49, *p* < 0.001) and 38.5% (F_3,20_ = 35.47, *p* < 0.001) of control mice, respectively, and increased latency time to 486.7% (F_3,20_ = 15.50, *p* < 0.001) of control mice (Figure 6A–C). However, oral administration of NK109 and NS improved spontaneous alteration in Tg mice to 80.5% and 89.4% of control mice, respectively, exploration in Tg mice to 58.0% and 62.0% of control mice, respectively, and latency time in Tg mice to 230.1% and 173.0% of control mice, respectively. The cognition-improving potency between NK109 and NS was not significantly different. Tg mice exhibited increased p-p65/p65, Psen-1, Aβ, TNF-α, and IL-1β expression and NF-κB^+^Iba1^+^ cell (activated microglia) population compared with those in the control, while p-CREB/CREB, claudin-5, and IL-10 expression and BDNF^+^NeuN^+^ cell population increased (Figure 6D–J). In the immunoblotting analysis, they increased p-CREB/CREB and claudin-5 expression, while Aβ, Psen-1, and p-p65/p65 expression were decreased (Figure 6K).

**Figure 7:**
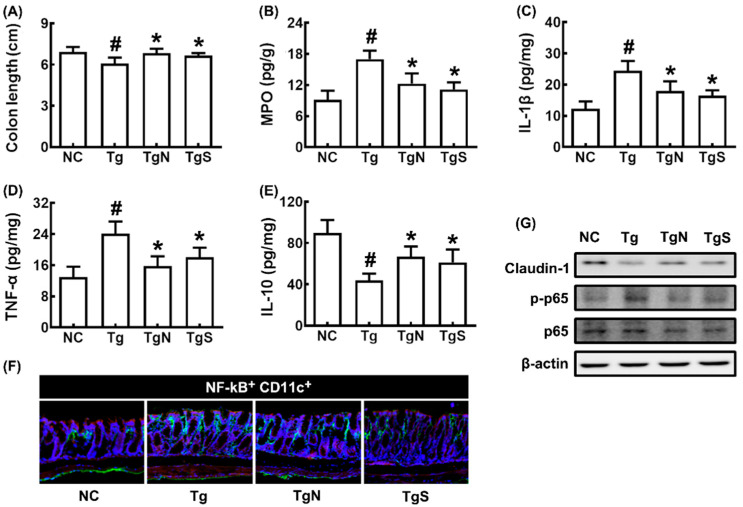
Effects of NK109 and its supplement NS on CI in 5xFAD transgenic (Tg) mice. Effects on colon length (**A**) and myeloperoxidase (MPO) (**B**), IL-1β (**C**), TNF-α (**D**), and IL-10 (**E**) expression in the colon. (**F**) Effects on NF-κB^+^CD11c^+^ cell population in the colon. (**G**) Effects on the expression of claudin-1, p-p65, p65, and β-action. NC, normal control mice; Tg, treated with saline in Tg mice; TgN, treated with NK109 in Tg mice; TgS, treated with NS in Tg mice. Data values are indicated as mean  ±  SD (*n* = 7). ^#^ *p*  <  0.05 vs. Nc. * *p*  <  0.05 vs. Tg.

In Tg mice, colon shortening and colonic myeloperoxidase, TNF-α, and IL-1β expression and NF-κB+CD11c+ cell number increased more strongly than in control mice, while IL-10 and claudin-1 expression decreased (Figure 7A–F). However, oral administration of NK109 or NS decreased myeloperoxidase, TNF-α, and IL-1β expression and NF-κB-postive cell populations in the colon of Tg mice, while claudin-1 and IL-10 expression increased.

**Figure 8:**
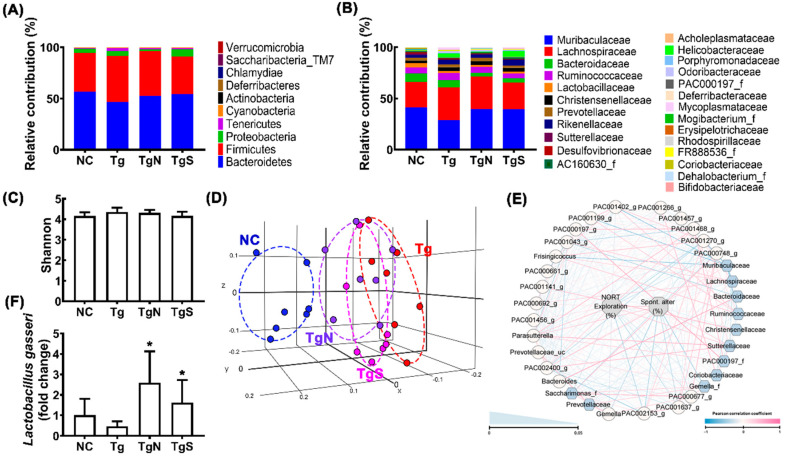
Effects of NK109 and NS in the gut microbiota composition in Tg mice. Effects on the composition at the phylum level (**A**) and family level (**B**). Effects on the α-diversity (OTU richness, c) (**C**), β-diversity (principal coordinate analysis plot based on Jensen–Shannon analysis) (**D**), and network of differentially enriched gut microbiota in volunteers with MCI (**E**). (**F**) Effects on the NK109 population. * *p*  <  0.05 vs. Tg.

Next, we tested the effects of NK109 and NS on the gut microbiota composition in Tg mice (Figure 8, supplement Tables S7–S9). Tg mice exhibited a higher abundance of Proteobacteria and Tenericutes populations than the control mice (Figure 8A,B). Moreover, the β-diversity of Tg mouse fecal microbiota was different to those of the control mice, while the α-diversity was not different (Figure 8C,D). However, oral administration of NK109 or NS weakly shifted the β-diversity in Tg mice to that in control mice, while the α-diversity was not affected (Figure 8C,D). At the phylum level, treatment with NK109 or NS decreased Proteobacteria and Tenericutes populations in Tg mice, while Actinobacteria populations increased (Figure 8A, Supplementary Tables S8–S10). At the family level, NK109 or NS treatment increased Muribaculaceae and Rhodospiraceae populations, while Helicobacteriaceae, Porphyromonadaceae, and Mycoplasmataceae populations decreased (Figure 8B). In the network analysis, Muribaculaceae, Sutterellacease, and Coriobacteriaceae populations were positively associated with spontaneous alteration in the YMT or exploration in the NORT, while Gemella_f, Saccharimonas_f, and Christensenllaceae populations were negatively associated (Figure 8E, Supplementary Tables S11 and S12). Moreover, NK109 or NK treatment increased NK109 populations in the feces of Ag mice (Figure 8F).

## 4. Discussion

Gut microbiota bidirectionally communicates with the brain via the gut [7,13]. Excessive exposure to stressors causes CI and gut microbiota dysbiosis [9,29,30]. Stressor-induced anxiety/depression accelerates the occurrence of dementia through the HPA and MGB axes [31]. Ageing-involved chronic inflammation also stimulates dysregulated Aβ deposition in the brain and microbiota dysbiosis in the gut, which accelerate the occurrence of dementia. In addition, fecal microbiota transplantation from aged adults (mice) with CI causes CI in the transplanted mice [32,33,34]. Ag adults and Ag mice exhibit a higher abundance of gut Proteobacteria, including Escherichia coli [26]. Excessive exposure to Escherichia coli causes colitis and microbiota dysbiosis by the activation of the MGB axis, resulting in CI with neuroinflammation [35,36,37,38,39]. In the present study, FMT from volunteers with MCI caused CI-like behaviors and induced neuroinflammation and colitis in mice. Moreover, the β-diversities of gut microbiota in Ag and Tg mice were significantly different to those of healthy Yg and control mice, respectively. These results support that dementia can cause the fluctuation of gut microbiota composition (gut microbiota dysbiosis), which can cause CI with neuroinflammation.

Here, we also found that probiotic NK109 and its supplement NS increased cognition-related behaviors in Ag, Tg, and MCF-transplanted mice. They down-regulated the expression levels of proinflammatory cytokines TNF-α and IL-1β and populations of NF-κB^+^Iba1^+^ cells in the hippocampus. However, they up-regulated the expression of BDNF and IL-10 and populations of BDNF^+^NeuN^+^ cells in the hippocampus and colon. Oral administration of B. longum NK46 or L. plantarum C29 increases cognitive decline in Ag and Tg mice by inducing NF-κB-mediated BDNF expression [15,17]. L. plantarum TWK10 also alleviates aging-related CI in mice by regulating gut microbiome [40]. Lactobacillus fermentum JDFM216 also alleviates cognitive behavior in aged mice [41]. Other lactobacilli and bifidobacteria improve cognitive decline in Ag mice [42]. A probiotic mixture also improves cognitive deficit by the inhbition of NF-κB signaling and inflammatory responses [43]. These findings suggest that NK109 and NS may reduce neuroinflammation by regulating the expression of proinflammatory cytokines and antiinflammatory cytokines and thereafter increasing cognitive function by inducing BDNF expression.

NK109 and NS also suppressed TNF-α, IL-1β, myeloperoxidase expression levels and NF-κB^+^CD11c^+^ cell numbers in the colon of Ag, Tg, and MCF-transplanted mice. Lee et al. repored that B. longum NK46 also suppressed TNF-α levels and NF-κB^+^CD11c^+^ cell populations in the colon and blood of Ag and Tg mice [15]. Lactobacillus acidophilus suppresses TNF-α and IL-1β expression in mice with traumatic brain damage [44]. Probiotics modulate the MGB axis and ameliorate cognitive deficits in Ag SAMP8 mice [44]. Probiotics also improve cognitive function in Ag mice by modulating gut microbiota [45]. We reported that NK109 alleviated TNF-α levels in the colon and blood of mice with *E. coli* K1-induced CI [19]. These results suggest that NK109 and NS alleviated colitis by suppressing NF-κB-mediated inflammatory signaling, resulting in the suppression of systemic inflammation.

NK109 and NS alleviated gut microbiota dysbiosis in Ag and Tg mice. In particular, they modulated CI-involved gut microbiota in the network analysis. Thus, Lachnospiraceae and Rikenellaceae populations were positively associated with spontaneous alteration in the YMT and exploration in the NORT in Ag mice, while Gemella_f, Saccharimonas_f, and Christensenllaceae populations were positively associated in Tg mice. However, oral administration of NK109 and NS all shifted the β-diversities of gut microbiota to healthy Yg and control mice in Ag and Tg mice, respectively. Saccharomyces boulardii alleviates ampicillin-induced gut dysbiosis and cognitive decline in mice [46]. Lactobacillus rhamnosus and B. longum mitigates ampicillin- or cyclophosphamide-induced gut dysbiosis and CI in mice [47]. These findings suggest that NK109 and NS can alleviate CI-induced gut microbiota dysbiosis.

The cognitive function-improving efficacy of NS was slightly, but not significantly, higher than that of NK109. SE is known to be able to alleviate CI. These findings suggest that the supplement of SE in NK109 may additively alleivate CI in vivo.

## 5. Conclusions

FMT from patients with MCI caused CI and neuroinflammation in mice, resulting in a useful MCI animal model. NK109 and its supplement NS may improve cognitive decline, neuroinflammation, and gut inflammation by modulating NF-κB-mediated BDNF expression and gut microbiota. Gut microbiota dysbiosis-alleviating probiotics may be beneficial for the therapy of CI and systemic inflammation.

## Data Availability

The datasets used and/or analyzed during the current study are available from the corresponding author on reasonable request. Microbiota data are deposited in NCBI (accession number PRJNA 915132).

## Supplementary Materials

The following supporting information can be downloaded at: https://www.mdpi.com/article/10.3390/nu15030790/s1, Figure S1: title; Table S1: Clinical characteristics of study participants; Table S2: Table S2. Primers for NK109 and 16S rRNA gene; Table S3: Effects of NK109 and NS on the fecal microbiota composition at the phylum level in Ag mice; Table S4: Effects of NK109 and NS on the fecal microbiota composition at the family level in Ag mice; Table S5: Effects of NK109 and NS on the fecal microbiota composition at the phylum level in Ag mice; Table S6: The relationship between differentially enriched gut microbiota composition and spontaneous alteration score in YMT in Ag mice (in the network analysis); Table S7: The relationship between differentially enriched gut microbiota composition and exploration score in NORT in Ag mice (in the network analysis); Table S8: Effects of NK109 and NS on the fecal microbiota composition at the phylum level in Tg mice; Table S9: Effects of NK109 and NS on the fecal microbiota composition at the family level in Tg mice; Table S10: Effects of NK109 and NS on the fecal microbiota composition at the species level in Tg mice; Table S11: The relationship between differentially enriched gut microbiota composition and spontaneous alteration score in YMT in Tg mice (in the network analysis); Table S12: The relationship between differentially enriched gut microbiota composition and exploration score in NORT in Tg mice (in the network analysis). And Supplement File S1. Refs. [15,19,25,27,37] are cited in Supplementary Materials.
