# Supplementary material for: Lactobacillus gasseri NK109 and Its Supplement Alleviate Cognitive Impairment in Mice by Modulating NF-κB Activation, BDNF Expression, and Gut Microbiota Composition
Source: Nutrients. 2023 Feb 3;15(3):790. doi: 10.3390/nu15030790 (PMC9921112; doi:10.3390/nu15030790)
Supplement: Supplementary file 1 [file nutrients-15-00790-s001.zip › nutrients-2148118-supplementary tables.pdf]

Table S1. Clinical characteristics of study participants

Volunteer		Age (Gender)	CERAD-K	Body mass index[1)]	Medicines within 3 months[2)]
healthy	Mean±SD	60.0±6.6[3)]	75.3±1.5	23.9±1.4	
	Each	53 (F)<br>61 (F)<br>66 (M)	75<br>74<br>77	25.4<br>23.6<br>22.7	No<br>No<br>No
MCI	Mean±SD	65.3±4.2	59.3±6.4	26.0±3.3	
	Each	70 (M)<br>64 (F)<br>62 (F)	52<br>63<br>63	22.2<br>27.3<br>28.5	No<br>No<br>No

[1)] Body mass index (BMI) BMI is defined as the body mass divided by the square of the body height, and is universally expressed in units of kg/m^2, resulting from mass in kilograms and height in metres.
[2)] medications that can affect the gut microbiota, such as painkillers, anti-inflammatory drugs, and antibiotics for > 3 months. Acute gastrointestinal symptoms such as diarrhea and abdominal pain within the last week.
[3)] Indicated as mean ± SD.

Table S2. Primers for NK109 and 16S rRNA gene

	Primer	
	Forward	Reverse
NK109 gene	AGCGAGCTTGCCTAGATGAATTTG	TCT TTT AAA CTC TAG ACA TGC GTC
16S rRNA gene	TCGTCGGCAGCGTCAGATGTGTATAAG<br>AGACAGGTGCCAGCMGCCGCGGTAA	GTCTCGTGGGCTCGGAGATGTGTATAAG<br>AGACAGGGACTACHVGGGTWTCTAAT

Table S3. Effects of NK109 and NS on the fecal microbiota composition at the phylum level in Ag mice

Taxon Name	mean ± SD[1)]				
	Yg	YgS	Ag	AgN	AgS
Bacteroidetes	51.7±12.3	50.9±7.0	48.6±5.0	54.0±49.9	49.9±3.7
Firmicutes	43.4±12.0	42.6±6.9	46.6±5.3	40.9±42.9	42.9±4.5
Proteobacteria	2.7±1.7	2.0±0.2	3.4±0.8	2.7±2.9	2.9±1.3
Tenericutes	1.7±2.0	1.6±0.9	0.4±0.2	1.1±1.1	1.1±0.9
Actinobacteria	0.3±0.1	0.1±0.1	0.9±0.5*	0.4±0.5	0.5±0.3
Cyanobacteria	0.1±0.2	1.0±0.6	0.2±0.1	0.7±1.1	1.1±1.4
Deferribacteres	0.1±0.1	0.2±0.2	0.0±0.0	0.2±0.1	0.1±0.2
Saccharibacteria_TM7	0.0±0.0	0.0±0.0	0.0±0.0	0.0±0.0	0.0±0.0
Verrucomicrobia	0.0±0.0	1.5±1.3	0.0±0.0	0.0±1.4	1.4±1.9
Fusobacteria	0.0±0.0	0.0±0.0	0.0±0.0	0.0±0.0	0.0±0.0

[1)]Mean ± SD

Table S4. Effects of NK109 and NS on the fecal microbiota composition at the family level in Ag mice

Taxon Name	mean ± SD[1)]				
	Yg	YgS	Ag	AgN	AgS
Muribaculaceae	35.7±10.8	37.7±7.0	36.2±3.1	31.2±7.2	31.5±4.7
Lachnospiraceae	26.8±12.4	28.1±5.0	20.2±6.8	21.5±5.2	23.6±4.0
Lactobacillaceae	8.0±6.0	1.1±0.5	13.8±5.2	8.1±2.4	8.9±4.3
Rikenellaceae	6.3±2.3	3.0±1.3	2.0±0.3	2.6±1.3	4.7±1.2
Ruminococcaceae	6.0±2.4	9.5±2.3	6.2±2.3	7.1±1.9	7.3±2.6
Prevotellaceae	4.8±3.0	4.7±3.3	2.8±1.6	12.5±5.2	6.3±4.3
Bacteroidaceae	2.8±1.5	2.7±1.1	5.4±1.9	5.7±1.1	4.9±1.5
Christensenellaceae	1.9±0.5	2.7±0.5	2.4±0.7	1.9±1.2	1.5±1.3
Odoribacteraceae	1.6±1.3	1.7±0.6	1.2±0.4	0.6±0.3	1.9±1.3
Acholeplasmataceae	1.5±2.1	1.4±1.0	0.2±0.1	0.9±0.8	0.7±0.8
Helicobacteraceae	1.0±1.7	0.1±0.1	1.2±0.9	0.3±0.2	1.3±1.6
Sutterellaceae	1.0±1.3	0.8±0.3	1.3±0.7	1.3±0.7	0.6±0.4
Desulfovibrionaceae	0.5±0.1	0.8±0.3	0.7±0.4	0.7±0.3	0.6±0.3
Erysipelotrichaceae	0.4±0.5	0.8±0.3	1.9±1.1	1.3±1.0	1.1±1.1
AC160630_f	0.3±0.1	0.7±0.4	0.6±0.3	0.7±0.8	0.5±0.3
Porphyromonadaceae	0.3±0.2	0.5±0.2	0.3±0.1	0.7±0.3	0.2±0.2
Coriobacteriaceae	0.2±0.1	0.1±0.1	0.3±0.1	0.2±0.1	0.5±0.3
Rhodospirillaceae	0.2±0.1	0.3±0.2	0.2±0.1	0.3±0.3	0.3±0.3
Dehalobacterium_f	0.2±0.1	0.3±0.1	0.2±0.1	0.2±0.1	0.2±0.1
PAC000197_f	0.1±0.3	0.2±0.2	0.1±0.1	0.2±0.1	0.4±0.4
FR888536_f	0.1±0.2	1.0±0.6	0.2±0.1	0.7±0.4	1.1±1.4
Deferribacteraceae	0.1±.1	0.2±0.2	0.0±0.0	0.2±0.1	0.1±0.2
Mogibacterium_f	0.1±0.0	0.1±0.0	0.1±0.0	0.1±0.0	0.1±0.0
Peptococcaceae	0.0±0.0	0.0±0.0	0.0±0.0	0.0±0.0	0.0±0.0
Bifidobacteriaceae	0.0±0.0	0.0±0.0	0.6±0.4*	0.2±0.2	0.0±0.0[#]

[1)]Mean ± SD

Table S5. Effects of NK109 and NS on the fecal microbiota composition at the phylum level in Ag mice

Taxon Name	mean ± SD[1)]				
	Yg	YgS	Ag	AgN	AgS
Lactobacillus murinus group	7.8±5.9	0.4±0.2	12.9±5.0	5.8±2.9	7.4±3.7
PAC001077_s	6.2±3.8	1.0±0.8	0.8±0.6	2.4±1.6	8.3±6.4
PAC001070_s group	6.1±5.7	5.9±1.5	8.6±2.2	5.8±4.3	4.2±3.8
PAC001093_s	3.5±5.4	0.3±0.7	0.5±1.0	0.1±0.2	2.6±3.9
PAC002450_s	3.0±4.1	10.4±1.8	5.6±3.1	4.4±3.6	1.1±2.5
PAC001060_s	2.5±1.4	0.1±0.2	0.4±0.3	0.4±0.7	1.4±0.8
PAC001383_s	2.4±2.8	0.1±0.1	0.1±0.1	0.7±0.9	0.7±1.1
KE159538_s	2.0±2.2	1.5±1.7	0.4±0.5	1.0±0.9	0.4±0.7
PAC001071_s	1.8±1.6	0.0±0.0	0.1±0.0	0.2±0.2	1.7±1.6
PAC002443_s	1.8±1.5	0.4±0.3	0.3±06	0.8±0.7	0.3±0.2
PAC002399_s	1.8±0.5	0.6±0.5	0.6±0.7	0.4±0.2	0.6±0.3
PAC002444_s	1.7±0.9	1.3±1.1	0.4±0.2	0.2±0.3	0.6±0.5
Muribaculum intestinale	1.6±1.6	1.0±0.9	2.8±0.6	2.3±1.4	0.4±0.2
PAC001080_s	1.5±2.1	1.4±1.0	0.2±0.2	0.9±0.8	0.6±0.8
PAC002400_s	1.4±1.1	1.2±0.4	0.7±0.2	0.6±0.3	1.0±0.6

PAC000198_s	1.4±1.2	0.6±0.7	0.4±0.8	0.2±0.1	0.4±0.3
PAC001065_s group	1.2±0.9	0.1±0.0	0.4±0.3	0.1±0.1	0.4±0.4
AB606328_s	1.1±1.3	0.9±0.3	0.4±0.2	0.4±0.2	1.1±0.8
KE159571_s	1.1±1.5	0.0±0.0	0.0±0.0	0.0±0.1	0.0±0.0
PAC002481_s	1.1±0.6	1.4±0.4	0.9±0.4	0.7±0.3	1.2±1.0
PAC001696_s	1.0±2.1	4.3±2.8	0.5±1.3	0.0±0.0	1.7±4.0
PAC001128_s	1.0±0.4	0.3±0.1	0.7±0.3	0.3±0.2	0.5±0.5
PAC001066_s	1.0±0.3	0.6±0.2	0.2±0.2	1.1±0.4	0.5±0.2
PAC001515_s	1.0±0.9	1.0±1.2	0.2±0.3	0.1±0.2	0.6±1.3
PAC002441_s group	0.9±0.6	0.6±0.3	0.5±0.1	0.6±0.2	0.5±0.2

[1)]Mean ± SD

Table S6. The relationship between differentially enriched gut microbiota composition and spontaneous alteration score in YMT in Ag mice (in the network analysis)

	Spontaneous alteration score in Y-maze task	R	R^2	P-value		Spontaneous alteration score in Y-maze task	R	R^2	P-value
Phylum	Bacteroidetes	0.1061	0.0113	0.5769	species	Lactobacillus murinus group	-0.5401	0.2917	0.0021
	Firmicutes	-0.1032	0.0107	0.5874		PAC002443_s	0.497	0.2470	0.0052
	Actinobacteria	-0.5982	0.3578	0.0005		PAC002444_s	0.3888	0.1512	0.0337
Family	Muribaculaceae	-0.1368	0.0187	0.4712		PAC001066_s	0.5784	0.3345	0.0008
	Lachnospiraceae	0.3368	0.1134	0.0688		PAC002447_s	-0.4414	0.1948	0.0146
	Lactobacillaceae	-0.5606	0.3143	0.0013		PAC001119_s	0.4182	0.1749	0.0215
	Rikenellaceae	0.4272	0.1825	0.0186		PAC001688_s group	0.4202	0.1766	0.0208
	Ruminococcaceae	0.3850	0.1482	0.0356		PAC001382_s	0.4007	0.1606	0.0282
	Bacteroidaceae	-0.3446	0.1187	0.0622		PAC001684_s	0.4827	0.2330	0.0069
	Odoribacteraceae	0.0286	0.0008	0.8807		PAC001666_s	0.4276	0.1828	0.0184
	Erysipelotrichaceae	-0.4636	0.2149	0.0099		PAC001558_s	-0.5133	0.2635	0.0037
	Coriobacteriaceae	-0.2678	0.0717	0.1525		PAC001668_s	0.3931	0.1545	0.0316
	Bifidobacteriaceae	-0.5992	0.3590	0.0005		PAC001267_s	-0.4165	0.1735	0.022
	Peptostreptococcaceae	-0.0633	0.0040	0.7399		KE159714_s group	-0.4344	0.1887	0.0164
Genus	Lactobacillus	-0.5609	0.3146	0.0013		Lactobacillus_uc	-0.6899	0.4760	<0.0001
	Alistipes	0.4534	0.2056	0.0119		AB599946_s	-0.5511	0.3037	0.0016
	LLKB_g	0.3416	0.1167	0.0647		PAC001199_s	0.3943	0.1555	0.0311
	PAC000186_g	-0.5416	0.2933	0.002		KB822463_s	-0.7004	0.4906	<0.0001
	Bacteroides	-0.3446	0.1187	0.0622		Bifidobacterium pseudolongum group	-0.6015	0.3618	0.0004
	Oscillibacter	0.4118	0.1696	0.0237		PAC001589_s	-0.5161	0.2664	0.0035
	PAC000664_g	-0.0399	0.0016	0.8343		PAC001266_s	-0.6567	0.4313	<0.0001
	KE159571_g	0.255	0.0650	0.1738		PAC001043_g_uc	-0.5801	0.3365	0.0008
	Odoribacter	0.02849	0.0008	0.8812		Lactobacillus intestinalis	-0.5801	0.3365	0.0008
	PAC001092_g	0.2355	0.0555	0.2104		PAC001396_s	-0.6622	0.4385	<0.0001
	PAC001112_g	-0.2443	0.0597	0.1933		PAC001294_s	-0.3945	0.1556	0.031
	PAC001066_g	0.5794	0.3357	0.0008		HQ740432_s	-0.4342	0.1885	0.0165
	PAC000692_g	0.1377	0.0190	0.4682		PAC001528_s	-0.4147	0.1720	0.0227
	Agathobaculum	0.1754	0.0308	0.3539		PAC002299_s	-0.4755	0.2261	0.0079
	Eubacterium_g17	0.3643	0.1327	0.0478		PAC001488_s group	-0.5447	0.2967	0.0019

	Acetatifactor	-0.0252	0.0006	0.895		Clostridium celatum group	-0.7739	0.5989	<0.0001
	PAC001043_g	0.3676	0.1351	0.0457		PAC001271_s	-0.493	0.2430	0.0056
	PAC001199_g	0.3059	0.0936	0.1002		Clostridium_uc	-0.7017	0.4924	<0.0001
	Clostridium_g21	-0.1248	0.0156	0.5111		DQ815552_s	-0.5526	0.3054	0.0015
	PAC000661_g	-0.0656	0.0043	0.7307		DQ815878_s	-0.6489	0.4211	0.0001
	Dubosiella	-0.06	0.0036	0.7527		FJ881246_s	-0.5212	0.2716	0.0031
	PAC001091_g	-0.3485	0.1215	0.0591		Lactococcus garvieae group	-0.7121	0.5071	<0.0001
	PAC001516_g	-0.0998	0.0100	0.6		PAC000686_s	-0.5168	0.2671	0.0035
	KE159605_g	-0.2771	0.0768	0.1383		PAC001090_s	-0.489	0.2391	0.0061
	PAC001266_g	-0.6565	0.4310	<0.0001		PAC001092_g_uc	-0.4146	0.1719	0.0227
	Bifidobacterium	-0.5992	0.3590	0.0005		PAC001553_s group	-0.5539	0.3068	0.0015
	PAC001116_g	-0.493	0.2430	0.0056		PAC001588_s	-0.6109	0.3732	0.0003
	Roseburia	-0.4205	0.1768	0.0207		PAC001665_s	-0.6387	0.4079	0.0001
	PAC001588_g	0.01566	0.0002	0.9345		PAC001902_s	-0.5875	0.3452	0.0006
	Lactococcus	-0.7439	0.5534	<0.0001					
	PAC001090_g	-0.489	0.2391	0.0061					

Table S7. The relationship between differentially enriched gut microbiota composition and exploration score in NORT in Ag mice (in the network analysis)

	Exploration score in NORT	R	R^2	P-value		Exploration score in NORT	R	R^2	P-value
Phylum	Bacteroidetes	0.1673	0.0280	0.3768	species	KE159538_s	0.4289	0.1840	0.018
	Firmicutes	-0.1879	0.0353	0.3202		PAC002443_s	0.4004	0.1603	0.0283
	Actinobacteria	-0.3981	0.1585	0.0293		PAC002399_s	0.4285	0.1836	0.0181
Family	Muribaculaceae	0.1490	0.0222	0.4319		PAC002444_s	0.4092	0.1674	0.0248
	Lachnospiraceae	0.1952	0.0381	0.3012		PAC002447_s	-0.3614	0.1306	0.0498
	Lactobacillaceae	-0.3830	0.1467	0.0367		PAC001119_s	0.3675	0.1351	0.0457
	Rikenellaceae	0.3502	0.1226	0.0578		PAC001688_s group	0.3897	0.1519	0.0333
	Ruminococcaceae	0.0721	0.0052	0.7052		PAC001382_s	0.3819	0.1458	0.0373
	Bacteroidaceae	-0.3706	0.1373	0.0438		PAC001684_s	0.4219	0.1780	0.0202
	Odoribacteraceae	0.0099	0.0001	0.9587		PAC001666_s	0.5377	0.2891	0.0022
	Erysipelotrichaceae	-0.2478	0.0614	0.1868		PAC001558_s	-0.4757	0.2263	0.0079
	Coriobacteriaceae	-0.2111	0.0446	0.2627		PAC001668_s	0.4798	0.2302	0.0073
	Bifidobacteriaceae	-0.3735	0.1395	0.0421		PAC001267_s	-0.3882	0.1507	0.034
	Peptostreptococcaceae	-0.2606	0.0679	0.1642		KE159714_s group	-0.5576	0.3109	0.0014
Genus	KE159538_g	0.348	0.1211	0.0595		Lactobacillus_uc	-0.4997	0.2497	0.0049
	Alistipes	0.3974	0.1579	0.0296		AB599946_s	-0.5164	0.2667	0.0035
	LLKB_g	0.3031	0.0919	0.1035		PAC001199_s	0.3779	0.1428	0.0395
	PAC000186_g	-0.3553	0.1262	0.054		KB822463_s	-0.6891	0.4749	<0.0001
	Bacteroides	-0.3706	0.1373	0.0438		Bifidobacterium pseudolongum group	-0.3713	0.1379	0.0434
	Oscillibacter	0.09756	0.0095	0.608		PAC001589_s	-0.4845	0.2347	0.0067
	PAC000664_g	-0.2359	0.0556	0.2095		PAC001266_s	-0.5561	0.3092	0.0014
	PAC001127_g	0.4285	0.1836	0.0181		PAC001043_g_uc	-0.5835	0.3405	0.0007
	KE159571_g	0.3751	0.1407	0.0411		Lactobacillus intestinalis	-0.4075	0.1661	0.0254
	Odoribacter	0.009914	0.0001	0.9585		PAC001396_s	-0.5827	0.3395	0.0007

	PAC001092_g	0.2075	0.0431	0.2713
	PAC001112_g	-0.1691	0.0286	0.3717
	PAC000692_g	0.08221	0.0068	0.6658
	Agathobaculum	0.2001	0.0400	0.2891
	Eubacterium_g17	0.4246	0.1803	0.0193
	Acetatifactor	-0.1031	0.0106	0.5877
	PAC001043_g	0.2268	0.0514	0.228
	PAC001199_g	0.3179	0.1011	0.0869
	Clostridium_g21	-0.1077	0.0116	0.5712
	PAC000661_g	-0.2457	0.0604	0.1905
	Dubosiella	0.05326	0.0028	0.7798
	PAC001091_g	-0.4245	0.1802	0.0194
	PAC001516_g	-0.1279	0.0164	0.5005
	KE159605_g	-0.3407	0.1161	0.0654
	PAC001266_g	-0.5905	0.3487	0.0006
	Bifidobacterium	-0.3735	0.1395	0.0421
	PAC001116_g	-0.451	0.2034	0.0124
	Roseburia	-0.6574	0.4322	<0.0001
	PAC001588_g	0.03653	0.0013	0.848
	Lactococcus	-0.6308	0.3979	0.0002
	PAC001090_g	-0.586	0.3434	0.0007

	PAC001294_s	-0.374	0.1399	0.0417
	HQ740432_s	-0.4601	0.2117	0.0105
	PAC002368_s	-0.4516	0.2039	0.0122
	PAC002299_s	-0.5816	0.3383	0.0007
	PAC001488_s group	-0.7246	0.5250	<0.0001
	Clostridium celatum group	-0.5599	0.3135	0.0013
	PAC001271_s	-0.6966	0.4853	<0.0001
	Clostridium_uc	-0.5086	0.2587	0.0041
	DQ815552_s	-0.5894	0.3474	0.0006
	DQ815878_s	-0.5101	0.2602	0.004
	FJ881246_s	-0.6017	0.3620	0.0004
	Lactococcus garvieae group	-0.6324	0.3999	0.0002
	PAC000686_s	-0.525	0.2756	0.0029
	PAC001090_s	-0.586	0.3434	0.0007
	PAC001092_g_uc	-0.4512	0.2036	0.0123
	PAC001553_s group	-0.4201	0.1765	0.0208
	PAC001588_s	-0.5462	0.2983	0.0018
	PAC001665_s	-0.6055	0.3666	0.0004
	PAC001902_s	-0.5269	0.2776	0.0028

Table S8. Effects of NK109 and NS on the fecal microbiota composition at the phylum level in Tg mice

Taxon Name	mean ± SD[1)]			
	NC	Tg	TgN	TgS
Bacteroidetes	56.7±8.5	46.5±7.2	52.8±10.5	54.2±6.3
Firmicutes	37.9±8.3	45.0±7.4	43.4±11.3	37.0±7.2
Proteobacteria	3.8±2.0	5.1±3.7	1.7±0.4	6.8±2.6
Tenericutes	1.0±0.6	2.9±2.1	1.4±2.1	1.5±2.4
Cyanobacteria	0.3±0.1	0.4±0.7	0.4±0.6	0.2±0.4
Actinobacteria	0.2±0.2	0.1±0.0	0.2±0.1	0.2±0.2
Deferribacteres	0.0±0.0	0.0±0.0	0.0±0.0	0.0±0.0
Chlamydiae	0.0±0.0	0.0±0.0	0.0±0.0	0.0±0.0
Saccharibacteria_TM7	0.0±0.0	0.0±0.0	0.0±0.0	0.0±0.0
Verrucomicrobia	0.0±0.0	0.0±0.0	0.0±0.0	0.0±0.0

[1)]mean ± SD

Table S9. Effects of NK109 and NS on the fecal microbiota composition at the family level in Tg mice

Taxon Name	mean ± SD[1)]			
	NC	Tg	TgN	TgS
Muribaculaceae	41.4±6.1	29.1±6.3	39.8±8.5	39.6±8.0
Lachnospiraceae	24.6±7.6	31.9±5.8	31.5±10.7	25.9±8.8
Bacteroidaceae	8.3±3.2	6.9±2.5	3.6±3.9	4.0±1.7
Ruminococcaceae	6.2±2.9	6.8±2.4	5.9±2.4	4.7±2.4

Lactobacillaceae	3.8±6.2	1.9±1.5	1.8±1.3	1.8±1.5
Christensenellaceae	2.8±1.1	4.0±2.4	3.9±1.9	4.0±1.7
Prevotellaceae	2.8±2.5	2.7±1.0	3.0±4.0	2.3±2.4
Rikenellaceae	2.5±0.9	4.5±1.6	3.5±1.9	5.6±3.7
Sutterellaceae	1.8±1.4	0.1±0.0	0.2±0.1	0.2±0.3
Desulfovibrionaceae	1.1±0.7	1.1±0.9	0.7±0.4	0.6±0.6
AC160630_f	0.7±0.2	0.7±0.6	1.0±0.4	1.4±0.8
Acholeplasmataceae	0.7±0.6	0.4±0.7	0.2±0.2	0.6±1.0
Helicobacteraceae	0.6±0.6	3.8±3.9	0.4±0.3	5.7±2.3
Porphyromonadaceae	0.6±0.4	0.4±0.2	0.0±0.0	0.2±0.3
Odoribacteraceae	0.4±0.5	2.1±1.9	2.0±2.3	1.1±0.6
PAC000197_f	0.3±0.5	0.0±0.0	0.2±0.3	0.0±0.1
FR888536_f	0.3±0.1	0.4±0.7	0.4±0.6	0.2±.4
Erysipelotrichaceae	0.2±0.2	0.2±0.1	0.1±0.1	0.3±0.5
Rhodospirillaceae	0.2±0.3	0.1±.2	0.4±0.3	0.4±0.5
Coriobacteriaceae	0.2±0.1	0.1±0.0	0.2±.1	0.1±.1
Dehalobacterium_f	0.1±0.1	0.2±.1	0.1±0.1	0.1±0.1
Bifidobacteriaceae	0.1±0.1	0.0±0.0	0.1±0.1	0.1±0.2
Mogibacterium_f	0.1±0.0	0.0±0.0	0.1±0.0	0.0±0.0
Mycoplasmataceae	0.0±0.0	2.3±1.8	1.0±1.9	0.8±1.9
Deferribacteraceae	0.0±0.0	0.0±0.0	0.0±0.0	0.0±0.0

[1)]mean ± SD

Table S10. Effects of NK109 and NS on the fecal microbiota composition at the species level in Tg mice

Taxon Name	AVE ± STD			
	NC	Tg	TgN	TgS
PAC001077_s	10.9±2.2	4.5±3.7	9.5±6.3	1.1±0.8
PAC002443_s	5.1±2.5	3.9±1.5	0.7±1.0	2.8±2.1
PAC001065_s group	4.7±1.5	2.6±1.6	3.2±2.2	3.2±2.7
PAC001770_s	3.3±3.0	0.0±0.0	1.8±3.8	1.5±2.9
PAC002400_s	3.3±1.0	0.7±0.8	1.1±0.6	1.6±1.1
KE159538_s	2.8±2.9	0.1±0.0	1.5±3.8	0.7±0.8
PAC001515_s	2.7±1.6	2.9±2.5	2.4±1.7	1.7±1.6
PAC001071_s	2.6±1.4	1.3±1.9	1.0±0.8	1.4±1.4
PAC001120_s	2.3±6.0	0.2±0.3	0.0v0.1	0.1±0.2
EF097112_s	2.1±1.0	0.7±0.3	1.0±0.7	1.6±1.1
Lactobacillus murinus group	2.0±2.9	1.7±1.3	1.0±0.8	1.2±1.2
PAC001066_s	1.9±0.4	1.8±2.3	1.0±0.9	1.1±0.9
PAC001060_s	1.9±0.7	1.9±1.7	1.9±2.4	2.8±2.3
Bacteroides_uc	1.8±0.9	0.2±0.1	0.1±0.1	0.1±0.2
PAC000198_s	1.7±0.6	1.0±0.7	1.8±1.2	1.9±1.4
PAC001064_s	1.6±1.0	2.3±1.8	3.8±2.7	3.1±1.1
PAC001070_s group	1.6±2.0	1.6±1.1	1.0±1.5	6.0±9.2
PAC001084_s	1.4±2.5	0.2±0.4	1.8±1.9	1.2±1.7
PAC001072_s	1.4±1.4	0.7±0.6	0.5±0.6	1.7±1.5
PAC001139_s	1.3±0.9	1.3±0.8	1.2±0.9	1.9±1.6
PAC002476_s	1.3±0.8	0.0±0.0	0.0±0.0	0.0±0.0
Bacteroides acidifaciens group	1.3±0.8	2.0±2.1	1.4±0.9	1.1±0.6
PAC002481_s	1.2±1.1	2.4±1.1	1.1±0.8	1.3±1.4
PAC001456_s	1.1±0.6	0.4±0.5	0.9±0.8	0.7±1.0

PAC001079_s	1.0±0.6	0.8±0.9	0.5±0.5	0.3±0.3

1)mean ± SD

Table S11. The relationship between differentially enriched gut microbiota composition and spontaneous alteration score in YMT in Tg mice (in the network analysis)

	Spontaneous alteration score in Y-maze task	R	R^2	P-value
Phylum	Bacteroidetes	0.3181	0.1012	0.0990
	Firmicutes	-0.2404	0.0578	0.2178
	Proteobacteria	-0.0182	0.0003	0.9270
	Tenericutes	-0.2355	0.0555	0.2277
	Actinobacteria	0.3721	0.1385	0.0512
	Saccharibacteria_TM7	-0.4306	0.1854	0.0222
Family	Muribaculaceae	0.4444	0.1975	0.0178
	Lachnospiraceae	-0.2747	0.0755	0.1571
	Bacteroidaceae	0.0604	0.0036	0.7601
	Ruminococcaceae	-0.0610	0.0037	0.7580
	Christensenellaceae	-0.2935	0.0861	0.1296
	Prevotellaceae	-0.1767	0.0312	0.3684
	Sutterellaceae	0.5582	0.3116	0.0020
	PAC000197_f	0.2403	0.0577	0.2181
	Coriobacteriaceae	0.2985	0.0891	0.1229
	Gemella_f	-0.5184	0.2687	0.0047
	Saccharimonas_f	-0.4306	0.1854	0.0222
Genus	Bacteroides	0.0584	0.0034	0.7680
	PAC002400_g	0.6329	0.4006	0.0003
	Prevotellaceae_uc	0.0106	0.0001	0.9573
	Parasutterella	0.5820	0.3387	0.0012
	PAC001456_g	0.3831	0.1468	0.0442
	PAC000692_g	0.1520	0.0231	0.4400
	PAC001141_g	-0.2329	0.0542	0.2330
	PAC000661_g	-0.0234	0.0005	0.9061
	Frisingicoccus	-0.0266	0.0007	0.8931
	PAC001043_g	-0.1783	0.0318	0.3641
	PAC000197_g	0.2411	0.0581	0.2164
	PAC001199_g	-0.1825	0.0333	0.3525
	PAC001402_g	-0.3866	0.1495	0.0421
	PAC001266_g	0.1457	0.0212	0.4596
	PAC001457_g	0.0304	0.0009	0.8780
	PAC001468_g	0.4060	0.1648	0.0321
	PAC001270_g	-0.4168	0.1737	0.0273
	PAC000748_g	-0.4412	0.1947	0.0188
	Gemella	-0.5184	0.2687	0.0047
	PAC000677_g	-0.4306	0.1854	0.0222
	PAC001637_g	-0.4639	0.2152	0.0129
	PAC002153_g	-0.5075	0.2576	0.0058
species	PAC002400_s	0.6245	0.3900	0.0004
	Bacteroides_uc	0.5648	0.3190	0.0017
	PAC002476_s	0.5814	0.3380	0.0012
	PAC002481_s	-0.3894	0.1516	0.0405
	PAC001456_s	0.3819	0.1458	0.0449
	PAC001358_s	0.3864	0.1493	0.0423
	HQ793361_s	0.6093	0.3712	0.0006
	EF603776_s	0.4596	0.2112	0.0139
	PAC002511_s group	-0.5051	0.2551	0.0061
	PAC001731_s	0.4060	0.1648	0.0321
	PAC001768_s	0.4596	0.2112	0.0139
	PAC002299_s	0.4519	0.2042	0.0158
	PAC001764_s	-0.3773	0.1424	0.0478
	PAC001495_s group	-0.3951	0.1561	0.0374
	PAC000688_s	-0.3909	0.1528	0.0397
	Bacteroides dorei	0.5531	0.3059	0.0023
	PAC001154_s	-0.6334	0.4012	0.0003
	EU505164_s	-0.5057	0.2557	0.0060
	PAC001781_s	0.3867	0.1495	0.0421
	Frisingicoccus_uc	-0.5762	0.3320	0.0013
	PAC001099_s	-0.4754	0.2260	0.0106
	PAC002420_s	-0.4537	0.2058	0.0153
	Gemella haemolysans group	-0.5184	0.2687	0.0047
	PAC001192_s group	-0.3906	0.1526	0.0399
	PAC001653_s	-0.3821	0.1460	0.0448
	PAC001787_s	-0.4440	0.1971	0.0179
	PAC002153_s	-0.5075	0.2576	0.0058
	PAC002422_s	-0.5093	0.2594	0.0056

Table S12. The relationship between differentially enriched gut microbiota composition and exploration score in NORT in Tg mice (in the network analysis)

	Exploration score in NORT	R	R^2	P-value
Phylum	Bacteroidetes	0.2102	0.0442	0.2831
	Firmicutes	-0.1214	0.0147	0.5384
	Proteobacteria	0.0054	0.0000	0.9783
	Tenericutes	-0.3845	0.1478	0.0433
	Actinobacteria	0.3078	0.0947	0.1111
	Saccharibacteria_TM7	-0.4154	0.1726	0.0279
Family	Muribaculaceae	0.2523	0.0637	0.1953
	Lachnospiraceae	-0.1743	0.0304	0.3750
	Bacteroidaceae	0.1836	0.0337	0.3497
	Ruminococcaceae	0.0451	0.0020	0.8199
	Christensenellaceae	-0.2286	0.0523	0.2419
	Prevotellaceae	-0.1688	0.0285	0.3906
	Sutterellaceae	0.6109	0.3732	0.0006
	PAC000197_f	0.3192	0.1019	0.0978
	Coriobacteriaceae	0.3231	0.1044	0.0935
	Gemella_f	-0.4055	0.1644	0.0323
	Saccharimonas_f	-0.4154	0.1726	0.0279
Genus	Bacteroides	0.1844	0.0340	0.3476
	PAC002400_g	0.6348	0.4030	0.0003
	Prevotellaceae_uc	0.0283	0.0008	0.8864
	Parasutterella	0.6831	0.4666	<0.0001
	PAC001456_g	0.4389	0.1926	0.0194
	PAC000692_g	0.1672	0.0280	0.3952
	PAC001141_g	-0.1723	0.0297	0.3805
	PAC000661_g	-0.1553	0.0241	0.4299
	Frisingicoccus	-0.0678	0.0046	0.7316
	PAC001043_g	-0.3756	0.1411	0.0489
	PAC000197_g	0.3158	0.0997	0.1016
	PAC001199_g	-0.2385	0.0569	0.2216
	PAC001402_g	-0.3639	0.1324	0.0570
	PAC001266_g	0.1598	0.0255	0.4167
	PAC001457_g	0.1120	0.0125	0.5704

	Exploration score in NORT	R	R^2	P-value
species	PAC002400_s	0.6382	0.4073	0.0003
	Bacteroides_uc	0.6273	0.3935	0.0004
	PAC002476_s	0.6972	0.4861	<0.0001
	PAC002481_s	-0.4039	0.1631	0.0331
	PAC001456_s	0.4433	0.1965	0.0181
	PAC001358_s	0.3771	0.1422	0.0479
	HQ793361_s	0.6164	0.3799	0.0005
	EF603776_s	0.5650	0.3192	0.0017
	PAC002511_s group	-0.5083	0.2584	0.0058
	PAC001731_s	0.5875	0.3452	0.0010
	PAC001768_s	0.4882	0.2383	0.0084
	PAC002299_s	0.5429	0.2947	0.0028
	PAC001764_s	-0.5019	0.2519	0.0065
	PAC001495_s group	-0.4497	0.2022	0.0164
	PAC000688_s	-0.4541	0.2062	0.0152
	Bacteroides dorei	0.5664	0.3208	0.0017
	PAC001154_s	-0.4262	0.1816	0.0237
	EU505164_s	-0.4099	0.1680	0.0303
	PAC001781_s	0.6781	0.4598	<0.0001
	Frisingicoccus_uc	-0.5330	0.2841	0.0035
	PAC001099_s	-0.4086	0.1670	0.0309
	PAC002420_s	-0.4114	0.1692	0.0296
	Gemella haemolysans group	-0.4055	0.1644	0.0323
	PAC001192_s group	-0.4133	0.1708	0.0288
	PAC001653_s	-0.4578	0.2096	0.0143
	PAC001787_s	-0.3904	0.1524	0.0400
	PAC002153_s	-0.4814	0.2317	0.0095
	PAC002422_s	-0.3816	0.1456	0.0451

	PAC001468_g	0.5875	0.3452	0.0010
	PAC001270_g	-0.4642	0.2155	0.0128
	PAC000748_g	-0.4298	0.1847	0.0224
	Gemella	-0.4055	0.1644	0.0323
	PAC000677_g	-0.4154	0.1726	0.0279
	PAC001637_g	-0.4554	0.2074	0.0149
	PAC002153_g	-0.4814	0.2317	0.0095

References

1. Yun, S.W.; Kim, J.K.; Lee, K.E.; Oh, Y.J.; Choi, H.J.; Han, M.J.; Kim, D.H. A Probiotic Lactobacillus gasseri Alleviates Escherichia coli-Induced Cognitive Impairment and Depression in Mice by Regulating IL-1β Expression and Gut Microbiota. *Nutrients* **2020**, *12*, doi:10.3390/nu12113441.
2. Lee, D.Y.; Shin, Y.J.; Kim, J.K.; Jang, H.M.; Joo, M.K.; Kim, D.H. Alleviation of cognitive impairment by gut microbiota lipopolysaccharide production-suppressing Lactobacillus plantarum and Bifidobacterium longum in mice. *Food Funct* **2021**, *12*, 10750-10763, doi:10.1039/d1fo02167b.
3. Lee, H.J.; Lee, K.E.; Kim, J.K.; Kim, D.H. Suppression of gut dysbiosis by Bifidobacterium longum alleviates cognitive decline in 5XFAD transgenic and aged mice. *Sci Rep* **2019**, *9*, 11814, doi:10.1038/s41598-019-48342-7.
4. Jang, H.M.; Lee, H.J.; Jang, S.E.; Han, M.J.; Kim, D.H. Evidence for interplay among antibacterial-induced gut microbiota disturbance, neuro-inflammation, and anxiety in mice. *Mucosal Immunol* **2018**, *11*, 1386-1397, doi:10.1038/s41385-018-0042-3.
5. Kim, J.K.; Choi, M.S.; Jeong, J.J.; Lim, S.M.; Kim, I.S.; Yoo, H.H.; Kim, D.H. Effect of Probiotics on Pharmacokinetics of Orally Administered Acetaminophen in Mice. *Drug Metab Dispos* **2018**, *46*, 122-130, doi:10.1124/dmd.117.077222.
