# Supplementary material for: Lactobacillus gasseri NK109 and Its Supplement Alleviate Cognitive Impairment in Mice by Modulating NF-κB Activation, BDNF Expression, and Gut Microbiota Composition
Source: Nutrients. 2023 Feb 3;15(3):790. doi: 10.3390/nu15030790 (PMC9921112; doi:10.3390/nu15030790)
Supplement: Supplementary file 1 [file nutrients-15-00790-s001.zip › nutrients-2148118-Supplementary File 1.pdf]

Supplement Materials and Methods

Volunteers

All volunteers were enrolled if antibacterial medications were not received within 3 months before stool collection (IRB No., KMC-201812004 and KMC-202103102, described detailedly in Supplement Materials and Methods). The study protocol and informed consent forms for the stool collection were approved by the Committee for the Care and Use of Clinical Study of the Medical School of Kyung Hee University (IRB No., KMC-201812004 and KMC-202103102). All experiments were performed in accordance with relevant named guidelines regulations. We confirmed that informed consent was obtained from all participants before the initiation of the study and that all methods involving human participants were carried out in accordance with the ethical principles of the Declaration of Helsinki and the Korean Good Clinical Practice guidelines.

Behavioral tasks

The YMT was carried out in a three-arm (120°) horizontal maze consisted of 40-cm-long and 3-cm-wide with 12-cm-high walls [1]. A mouse was initially placed within one arm and the sequence and sequence of arm entries were recorded for 8 min. A spontaneous alternation was indicated as entries into all three arms on consecutive choices and calculated as the ratio (%) of spontaneous to possible alternations.

The NORT was carried out in an open field box (45 × 45 × 45 cm) made by black acrylic panel [2]. The first trial was conducted in the box containing two identical marbles and each object-touching frequency was recorded for 10 min. On the next day, the second trial was conducted in the box containing one marble used in the first trial and a new marble. Exploration (%) was calculated as the ratio of time touching a new object to time touching both old and new marbles.

The BMT was carried out in the circular platform maze (diameter, 89 cm) with 20 holes (diameter, 5 cm) including an escape box 24, as previously reported [1,2]. The training/acquisition phase finished after mouse entered the escape box within 5 min was allowed to stay in the box for 30 s. If the mouse failed to enter the escape box within 5 min, it was deliberately led to the escape box for 30 s. Escape latency time was measured daily (two trials per day) for 4 consecutive days.

Immunblotting and enzyme-linked immunosorbent assay (ELISA)

The brain and colon tissues were lysed in radio immunoprecipitation assay lysis buffer (Pierce) containing a phosphatase inhibitor cocktail and a protease inhibitor cocktail at 4°C and centrifuged (10,000 *g*, 4°C, 10 min).

For the immunoblotting, the supernatants of the colon and cultured cell homogenates were subjected to sodium dodecyl sulfate-polyacrylamide gel electrophoresis and transferred to nitrocellulose membrane [3]. Proteins [BDNF, p16, p65, p-p65, CREB, p-CREB, Presenilin (Psen)-1, amyloid-β (Aβ), claudin-5, claudin-1, and β-action] were probed with antibodies, detected with horseradish peroxidase-conjugated secondary antibodies, and visualized with ECL detection kit.

For the ELISA of IL-1β, IL-10, and TNF-α, their levels were determined in the supernatant using their commercial ELISA kits (Ebioscience) [3].

Immunofluorescence assay

Immunofluorescence assay was performed, as previously reported [4]. Briefly, transcardially perfused brain and colon tissues were sectioned and washed with phosphate-buffered saline, blocked with normal serum, incubated with primary antibodies for NeuN, BDNF, NF-κB, LPS, Iba1, and/or CD11c overnight and secondary antibodies conjugated with Alexa Fluor 594 (Invitrogen) or Alexa Fluor 488 (Invitrogen) for 2 h. Nuclei were stained with 4',6-diamidino-2-phenylindole, dihydrochloride (DAPI). Immunostained sections were observed using a confocal microscope.

Quantitative real-time polymerase chain reaction (qPCR)

qPCR for NK109 was performed on the Rotor-Gene Q® thermocycler using DNA polymerase and SYBR Green I (Takara Bio Inc.: RR820A), as previously reported [5]. according to . Bacterial DNA was extracted from the feces using a QI-Aamp DNA stool mini kit. PCR amplification reaction was carried out as follows: initial denaturation at 95°C for 30 s, followed by 45 cycles of denaturation at 95°C for 5 s, annealing at 55ºC for 30 min, and extension at 72°C for 30 s. Primers for qPCR are indicated in Supplementary Table S2.
